# Physician Specialty, Experience, Region, Gender, and the Socioeconomic Status of Practice Location Predict Cost of Skin Cancer Procedures Performed in the Medicare Population

**DOI:** 10.7759/cureus.94940

**Published:** 2025-10-19

**Authors:** Justin A Freking, Brad R Woodie, Alan B Fleischer

**Affiliations:** 1 Department of Dermatology, University of Cincinnati College of Medicine, Cincinnati, USA

**Keywords:** demography, dermatologic surgical procedures, dermatology, health expenditures, health services research, medicare part b, skin neoplasms

## Abstract

Introduction: Skin cancer affects approximately five million individuals annually in the United States, with nonmelanoma skin cancers (NMSC) and melanoma amounting to $8.9 billion in annual healthcare costs.

Objective: Given the growing financial burden on the Medicare population, the present study aims to identify cost predictors to help detect outlier physician behavior and reduce healthcare costs.

Materials and methods: Using Medicare payments from 2017-2021, we analyzed malignant destructions and excisions (collectively, “removals”) and cutaneous closures on the trunk, arms, and legs. We included physicians from seven specialties and analyzed physician gender, years of experience, region, and socioeconomic status indicators. We subdivided physicians into three specialty subgroups, dermatologists, surgeons, and generalists, and characterized their overall case volume and costs. Then, adjusting for procedure type and volume, we calculated differences between actual and database-wide expected procedural costs and used k-means clustering to group physician cost patterns.

Results: Prior to adjustments, dermatologists billed higher-than-expected for removals, while surgeons had higher-than-expected total closure costs. However, with subsequent weighted adjustments, cluster analysis identified four unique physician cost phenotypes. Clustering demonstrated male physician gender, more experience, and practice in the Northeast, West, and affluent areas predicted high costs, while lower costs were associated with dermatologists and Midwestern practitioners. Between the highest and lowest cost clusters, the summed cost for a removal and closure differed by up to $1,735.

Conclusion: Physician and practice characteristics predict substantial cost differences, highlighting the need to understand these variations to optimize cost-effective skin cancer care.

## Introduction

Skin cancer, including melanoma and nonmelanoma skin cancers (NMSC), is the most prevalent cancer in the United States. Annually, there are an estimated 5.4 million cases of NMSC and over 100,000 new melanoma cases in the United States population [1,2]. The economic burden of skin cancer is substantial. From 2016 to 2018, the average annual expenditures for skin cancer treatment were $8.9 billion [3].

Standard excision, destructive modalities, and Mohs micrographic surgery are common treatments for skin cancer. Following excision, some lesions may require closure of varying complexity and cost. In recent years, the number of skin cancers treated by numerous specialists has increased, with otolaryngologists and plastic surgeons incurring the highest median costs for their procedures [4-7]. The increasing cost of NMSC care poses a significant challenge to Medicare, underscoring the need for cost-effective management strategies.

The present study examines the characteristics of physicians and their practices that may influence behavior in skin cancer treatment. The primary objective is to identify factors predicting higher costs for excision, destruction, and closure procedures of malignant skin lesions, excluding Mohs micrographic surgeries. We hypothesized that physician demographics and practice characteristics predict cutaneous cancer cost variability independent of case volume and procedure selection. A secondary objective was to characterize the cost phenotypes of cutaneous procedures performed by physicians of different specialties using k-means cluster analysis. As demonstrated by the American College of Mohs Surgery’s Improving Wisely initiative [8], understanding physician behavior can elucidate opportunities for enhancing performance. Therefore, identifying predictors of procedural expenditures may help guide strategies for optimizing cost and quality in skin cancer treatment, ultimately reducing healthcare expenditures for millions of patients.

## Materials and methods

Data collection and correction

Insurance claims for cutaneous procedures on the trunk, arms, and legs submitted by physicians across various specialties were obtained from the Medicare Provider and Service Database for the years 2017-2021 [9]. Such claims represent a physician’s annual caseload and payment amounts received from Medicare and thus are deidentified, eliminating concerns for patient confidentiality. We excluded procedures on the face, hands, feet, and genitalia due to increased cosmetic sensitivity and varying closure complexity. As our primary aim was to compare similar treatment approaches across specialties, we also excluded Mohs micrographic surgeries due to the limited number of non-dermatologists trained in the technique. Furthermore, physicians may select tumors for Mohs surgery due to increased risk [10], potentially resulting in more involved and higher-cost closures for trained Mohs surgeons. Nonetheless, non-Mohs procedures performed by micrographic dermatologic surgery specialists were included.

Current Procedural Terminology (CPT) codes were used to identify certain procedures. Specifically, we considered malignant destructions and malignant excisions, collectively referred to as “removals,” of all sizes on the trunk, arms, and legs. CPT codes for these excisions subsume a simple closure. Therefore, for cutaneous closures, we examined the more advanced types: intermediate, complex, tissue rearrangements (flaps), and full-thickness skin grafts. Importantly, we included only the smallest size range for each modality in order to avoid overrepresenting the costs of specialists who primarily perform large, complex repairs. Furthermore, split-thickness grafts were excluded due to a limited sample size and their major usage in treating patients with burns [7].

Closures can be performed following benign and malignant excisions. To match the relative complexity and size of the closures, we totaled benign excisions measuring 1 cm or less. This total was subtracted first from the total for intermediate closures, then from complex closures, and finally from grafts. Importantly, flaps were not adjusted because billing for a flap includes the excision. This corrected number of total services for closures was used from here on.

Sentinel lymph node biopsies and lymphadenectomies are commonly performed following excision to stage and care for patients with melanoma and advanced squamous cell carcinoma (SCC) [11,12]. Therefore, a physician who performs such procedures is likely to be a specialist in more complex or invasive tumors. As these procedures are not directly comparable to the majority of studied procedures, physicians with more than 10 instances of sentinel lymph node biopsies were excluded. This cutoff was selected in accordance with the Medicare policy that requires at least 11 reported services for a CPT code to be individually reflected in the public database.

Lastly, we included physicians from the following seven specialties, each with at least 40 physicians who had greater than 10 instances of the aforementioned procedures: dermatology, family practice, general surgery, internal medicine, micrographic dermatologic surgery (Mohs surgery), otolaryngology, and plastic and reconstructive surgery (plastic surgery).

Quantifying the difference between actual and expected costs

To account for variation in the frequency of a physician’s selection of size and procedure type of cutaneous procedures, cost differences were calculated as weighted means by procedure and size categories. For removals, six categories were created by separating by destructions and excisions and by the following size criteria: lesions less than or equal to 1 cm, lesions greater than or equal to 1 cm and less than or equal to 3 cm, and lesions greater than 3 cm. Closures were similarly separated by procedure type into four categories: intermediate, complex, flap, and graft closures.

“Cost difference” was calculated as the difference between a physician’s actual costs and expected costs. For a given treatment, the expected cost represented the pooled dataset’s weighted mean cost; a physician’s mean payment from Medicare was used for actual costs. The differences between actual and expected costs for each category were summed and then divided by a physician’s net total removals (to quantify a “removal cost difference”) or closures (for “closure cost difference”) using the following equation:

$Cost \, Difference=\frac{&sum;((AC_X*TS_X)-(MC_X*TS_X )) + ((AC_Y*TS_Y)-(MC_Y*TS_Y ))+(...))}{TS_{Overall}}$

Here, AC is the actual cost, and MC is the mean cost. TS signifies total services, and TS_Overall_ represents a physician’s total services for removals or closures. X and Y represent different categories of size and procedure.

Additionally, a subset of physicians in the dataset had malignant excision claims but no closure codes. Accordingly, we estimated an “implied cost difference” to account for these physicians’ potential uncoded simple closures or secondary intention healing. In the absence of total service or cost data, we calculated the weighted mean of the overall cost of closures across all modalities ($1236) and subtracted this from the physicians’ actual closure cost ($0) to arrive at an implied cost difference of -$1,236. Throughout, a positive cost difference indicates that a physician has higher-than-expected costs for a given procedure.

Physician and practice covariates

Several characteristics of physicians and their practice were examined. First, “years of experience” was calculated using the following equation:

$Years \, of \, Experience=2021-(Medical \, School \, Grad \, Year+Length \, of \, Residency)$

Medical school graduation years were obtained from the Medicare Doctors and Clinicians national dataset [13]. To estimate the length of residency, the standard number of years in training for each specialty was used [14]. 2021 was selected because it was the most recent year from which data were obtained.

Next, county codes were used to assign physicians to one of four regions (Midwest, Northeast, South, and West) according to the United States (US) Census Bureau classifications [15]. Each physician’s practice zip code was matched to the social deprivation index (SDI) from the American Community Survey and the median household income (MHI) from the US Census Bureau [16,17]. Both metrics served as proxies for socioeconomic status (SES); high MHI and low SDI correlate with higher SES.

Finally, we used specialty behavioral trends to group subspecialties in order to increase the sample size for inter-specialty comparison. For removals, we quantified physician procedural selection behavior, as we have done previously, by calculating a physician’s likelihood of performing an excision over a destruction (proportion of excisions, POE) [18]. For closures, we determined the proportion of a physician’s cases in which a complex repair, flap, or graft was selected over a simpler modality (proportion of advanced repair, POAR). The mean of individual POE and POAR values was then calculated for each specialty to represent overall procedural complexity.

Based on defined cutoffs for this behavioral likelihood value, physicians within each of the seven specialties were assigned to one of three subgroups. “Dermatology” (including only dermatologists) had the lowest mean of POE and POAR of 0.44. “General” (including family medicine and internal medicine) had a moderate mean between 0.54 and 0.57. “Surgery” (including general surgery, otolaryngology, Mohs surgery, and plastic surgery) had the highest mean of greater than 0.60. For each specialty subgroup, our initial analysis examined total procedural services and costs.

K-means cluster analysis

K-means clustering is a nonhierarchical method that groups observations into clusters by assigning each observation to a cluster that minimizes its distance from the cluster’s center. Cluster analysis was conducted on the removal cost difference and the closure cost difference. The optimal number of clusters was determined using the elbow method [19], which identifies the point at which increasing the cluster count (k) provides diminishing returns in decreasing the distance between cluster datapoints. This method yielded optimization with four clusters; thus, the k-means clustering procedure was run with k=4 on SAS 9.4 (SAS Institute, Cary, NC, USA).

After clustering, the centroid, the mean value of a cluster from which individual datapoint distance is calculated to determine cluster membership, was collected for each cluster’s removal and closure costs. We used the sum of a given cluster’s removal and closure centroid values to calculate an overall procedural cost. Then, we subtracted this value from the dataset’s mean procedural cost, which was standardized to $0, to derive a procedural cost difference. Negative values represented a cheaper-than-average removal followed by coded closure.

Statistical analysis

Statistical analysis was conducted to compare each cluster according to the above covariates. Welch’s one-way ANOVA and Tukey-Kramer post-hoc tests were performed on continuous variables; significance was set at α=0.05. Similarly, chi-square tests or Fisher’s exact tests with post-hoc Bonferroni adjustments were run on categorical variables, with an adjusted significance of α=0.008.

For continuous values, effect size is represented by η^2^, with the following threshold values: 0.01 for small, 0.06 for medium, 0.14 for large effect [20]. For categorical variables, effect size is represented by Cramer’s V, with the following thresholds: 0.05 for small, 0.10 for medium, and 0.15 for large effect [21]. All statistical analyses were once more conducted using SAS 9.4 (SAS Institute, Cary, NC, USA).

## Results

Using 2017-2021 Medicare data, select CPT codes were analyzed based on the aforementioned procedural type, size, and location inclusion criteria (Table 1).

**Table 1:** Current Procedural Terminology (CPT) codes included in analysis and their corresponding procedure type, size, and anatomical location. * Scalp closures are billed in the trunk, arms, and legs grouping, whereas scalp removals are billed in the neck, hands, feet, and genitalia grouping.

Type	Size	Location	Codes
Benign excision	≤1 cm	Trunk, arms, legs	11400, 11401
Benign excision	1.1-3 cm	Trunk, arms, legs	11402, 11403
Benign excision	>3 cm	Trunk, arms, legs	11404, 11406
Malignant excision	≤1 cm	Trunk, arms, legs	11600, 11601
Malignant excision	1.1-3 cm	Trunk, arms, legs	11602, 11603
Malignant excision	>3 cm	Trunk, arms, legs	11604, 11606
Intermediate closure	≤2.5 cm	Trunk, arms, legs, scalp*	12031
Complex closure	≤2.5 cm	Trunk, arms, legs, scalp	13100, 13120
Flap closure	≤10 cm^2^	Trunk, arms, legs, scalp	14000, 14020
Graft closure	≤20 cm^2^	Trunk, arms, legs, scalp	15200, 15220
Malignant destruction	≤0.5 cm	Trunk, arms, legs	17260
Malignant destruction	0.6 cm - 1.0 cm	Trunk, arms, legs	17261
Malignant destruction	1.1 cm - 2.0 cm	Trunk, arms, legs	17262
Malignant destruction	2.1 cm - 3.0 cm	Trunk, arms, legs	17263
Malignant destruction	3.1 cm - 4.0 cm	Trunk, arms, legs	17264
Malignant destruction	>4.0 cm	Trunk, arms, legs	17266
Lymph node biopsy	N/A	Inguinal nodes	38500
Lymph node biopsy	N/A	Cervical nodes	38510, 38520
Lymph node biopsy	N/A	Axillary nodes	38525
Lymphadenectomy	N/A	Cervical nodes	38720, 38724
Lymphadenectomy	N/A	Axillary nodes	38740, 38745
Lymphadenectomy	N/A	Inguinal nodes	38760, 38765

A comparison of total services and costs for destruction, excision, and closure codes revealed substantial differences among specialties in treating skin cancer. Despite making up 86% of the 8,943 physicians in the dataset, dermatologists represented 93% of the services and 93% of the removal cost totals. Conversely, 13% of surgeons in the dataset had removal services totaling 6% and removal costs totaling 6%, but they had closure services totaling 27% and closure costs totaling 23%. Furthermore, dermatologists had per-procedural costs for removals and closures $1 and $16 above the mean, respectively, across more than two and a half million procedures (Table 2).

**Table 2:** Summary of caseload and costs by physician specialty subgroup. * Please note the change in units and magnitude between table sections.

Variable	All physicians	Dermatology	Surgery	General
Physicians	8,943	7,695	1,106	142
Destructions (thousands^*^)	1,125	1,067	49	9
Excisions (thousands)	1,612	1,478	121	13
Closures (thousands)	179	128	48	4
Removal cost ($, millions^*^)	1,369	1,276	82	11
Closure cost ($, millions)	53	40	12	0.7
Price per removal ($^*^)	500	501	486	483
Price per closure ($)	295	311	259	190

**Figure 1:**
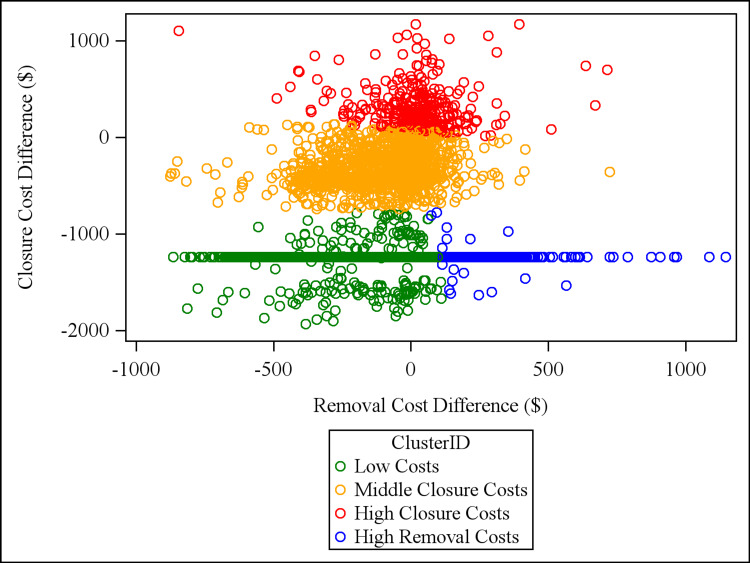
K-means cluster analysis of physicians plotted by the difference between actual and expected closure costs and the difference between actual and expected removal costs. Plotted values represent a physician’s removal and closure costs. Clusters are represented by different colored datapoints and were determined using k-means cluster analysis. Each point represents one physician.

Cluster analysis found four clusters of physicians with respect to the removal cost difference and the closure cost difference. The clusters were named according to cost phenotypes: “low costs,” “high removal costs,” “middle closure costs,” and “high closure costs.” We plotted each physician’s removal and closure costs, indicating cluster membership with different colors (Figure 1).

Covariates showed significant differences for specialty group, geographical region, gender, years of experience, MHI, and SDI between physicians in each cluster (Table 3).

**Table 3:** Physician and practice characteristics by removal cost and closure cost cluster membership. ^1^ Values for the four geographical regions, the three specialty subgroups, and physician gender represent the column frequencies of each categorical variable in a given cluster. For each of these variables, chi-square tests or Fisher’s exact tests with post-hoc Bonferroni adjustments were used to compare clusters, with adjusted significance defined as p < 0.008. Frequencies that do not share lowercase letters (^a^, ^b^, or ^c^) are therefore significantly different at α = 0.008. ^2^ Mohs surgery is micrographic dermatologic surgery; plastic surgery is plastic and reconstructive surgery. ^3^ 95% confidence intervals are shown in parentheses for the cluster means of the three continuous variables: years of experience, median household income, and social deprivation index. Welch’s one-way ANOVA and Tukey-Kramer post-hoc tests were used to compare groups with significance set at p < 0.05. Thus, means that do not share lowercase letters (^a^, ^b^, or ^c^) are significantly different at α = 0.05. ^4^ V represents effect size for categorical variables (0.10 > V ≥ 0.05 is a small effect), and η^2^ denotes effect size for continuous variables (0.06 > η^2^ ≥ 0.01 is a small effect).

Covariate		Low costs (n = 4639)	High removal costs (n = 322)	Middle closure costs (n = 1380)	High closure costs (n = 343)	Group difference and effect size
Geographical region^1^ (% in each cluster)	Midwest	19.4^a^	9.01^b^	20.5^a^	13.5^b^	p<0.0001; V=0.05^4^
	Northeast	16.4^a^	23.3^b^	14.7^a^	17.3^ab^	
	South	42.2^a^	38.8^a^	43.2^a^	44.4^a^	
	West	22.1^a^	28.9^b^	21.5^a^	24.9^ab^	
Dermatology^1^ (% of cluster with dermatology specialty)		85.7^a^	90.1^a^	82.0^b^	86.9^ab^	p=0.0003; V=0.05
Surgeons^1^ (% of cluster with general surgery, Mohs surgery^2^, otolaryngology, or plastic surgery specialty^2^)		12.7^a^	9.6^a^	16.5^b^	12.0^ab^	p=0.0005; V=0.05
Generalists^1^ (% of cluster with family practice or internal medicine specialty)		1.57^a^	0.31^a^	1.52^a^	1.17^a^	p=0.3; V=0.02
Physician gender^1^ (% male)		57.1^a^	69.3^b^	61.4^b^	66.8^b^	p<0.0001; V=0.07
Years of experience^3^		17.1^a ^(16.8-17.5)	27.4^b ^(26.1-28.7)	17.8^a ^(17.2-18.4)	22.4^c ^(21.4-23.5)	p<0.0001; η^2^=0.05^4^
Median household income of practice zip^3^		$75,063^a ^(74,173-75.950)	$88,624^b ^(84,443-92,807)	$75,487^a ^(73,703-77,271)	$80,751^c ^(77,346-84,157)	p<0.0001; η^2^=0.01
Social deprivation index of practice zip^3^		44.2^ab ^(43.4-45.0)	40.5^a ^(37.5-43.4)	44.9^b ^(43.5-46.4)	41.6^ab ^(39.9-44.3)	p=0.02; η^2^=0.00

Compared to the other clusters, the low-cost cluster had a higher proportion of dermatologists, female individuals, and less-experienced physicians practicing in areas in the Midwest and with low SES (i.e., low MHI, high SDI). Physicians in the high removal costs cluster had a higher proportion of dermatologists and the highest years of experience, SES, and number of physicians practicing in the Northeast and West. Physicians in the middle closure costs cluster had the highest percentage of surgeons and SDI, and lower years of experience and MHI. Lastly, physicians in the high closure costs cluster had moderate experience and MHI values.

**Figure 2:**
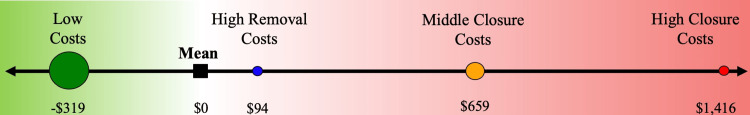
Cost for a removal and a closure from a mean physician in each physician cost cluster. The values represent each cluster’s sum of the mean value of removal cost difference and closure cost difference compared to the dataset mean, which is standardized to a value of $0. The circle sizes reflect each cluster’s sample size.

Finally, each cluster’s procedural cost differences, along with sample size, were plotted for comparison. “Low costs” had a procedural total $319 below the mean; “high removal costs” had a procedural total $1,416 above the mean (Figure 2).

## Discussion

This study characterized physician specialties that billed Medicare for non-Mohs skin malignancy removals and closures from 2017-2021. Previous studies have demonstrated cost differences between specialties in their treatment of NMSC [5,22]. In terms of total services and costs, dermatologists billed higher for removals and the highest per procedure. Conversely, surgeons billed higher-than-expected for closures but lower-than-expected per procedure. The difference in total costs between specialties closely matches the difference in their total service volumes. Thus, our data support prior studies that have shown dermatologists perform the majority of skin cancer procedures, with surgical specialties like plastic surgery performing a large number of more complex closures [4,6,7]. Unlike another previous study, we found that dermatologists had the highest unadjusted per-procedure costs [5]. Nonetheless, because of surgeons’ higher percentage of closure services, we support this study’s claim that complex case referrals to surgical specialists may drive up these physicians’ total closure costs and caseload [5].

In contrast to the initial unadjusted analysis, our subsequent cluster analysis, which involved weighted mean cost calculations, accounted for differences in case mix between physicians. Turning our cluster data into tangible costs borne by patients and Medicare revealed a vast difference between physicians. In particular, a removal followed by a closure could cost up to $1,735 more if performed by a “high closure costs” physician compared to a “low costs” counterpart. As annual Medicare payments to physicians for skin cancer treatments are already in the hundreds of millions and rising rapidly [4], this cost gap reflects one area where change could make a real economic impact by reducing insurance expenditures. Many of the sociodemographic factors studied are uncontrollable; however, treatment modality selection is one actionable factor that may help limit treatment costs.

Nevertheless, investigating cluster covariates permits identification of physician and practice factors associated with higher procedural costs. Accordingly, for closure costs, the “middle closure costs” cluster served as an excellent reference due to its large sample size and centrality. The “low costs” and “high removal costs” clusters, which included the lowest closure costs, had more dermatologists than the reference cluster. Additionally, these two clusters included all physicians who had no coded closures (and thus had only “implied cost differences”); interestingly, 87% of these physicians were dermatologists. Thus, dermatology predicts low closure costs, supporting a study that found that dermatologists are the most cost-effective specialists in performing minor cutaneous procedures [23]. Conversely, the “high closure costs” cluster had more experienced physicians, higher practice MHI, and fewer Midwestern physicians. Accordingly, greater experience and a wealthier practice location predict higher closure costs, while practice in the Midwest is less frequently associated with high closure costs.

For removal costs, comparison of the collective 74% of physicians in the “low costs” and “high removal costs” clusters shows that male physician gender, more experience, and practice in the Northeast, West, and in areas with high MHI are all predictive of high removal costs. Once again, Midwestern practice location predicts lower removal costs. We have previously shown that practicing in the Midwest is associated with the lowest odds of high outlier performance in Mohs procedures [24]. Here, we show that these protections extend to removal and closure costs. Contrary to our previous study on dermatologists’ removal behavior, which found that less experience was associated with outlier malignant excision performance, this study demonstrates that greater experience is instead a predictor of higher costs [18].

We note several limitations of the present study. In the Medicare dataset analyzed in this study, physicians must submit more than 10 instances of a specific CPT code annually to appear in the public dataset. By not reporting lower volumes, such a policy skews the analysis toward higher-volume physicians. Furthermore, physicians’ excisions are not matched to closures in this dataset, so certain specialists may see more complex removals or closures. Of note, definitions of intermediate and complex closures were revised on January 1, 2020, so physicians could show different practice patterns under the new criteria. An additional limitation of the Medicare Part B dataset is the omission of facility fees, which can be incurred in addition to CPT-coded removal and closure costs for hospital-based procedures. Furthermore, the type of malignancy being treated, the destruction modality, the depth of excision, the cosmetic outcome of closure, and patient-specific variables are unavailable in the data. Medicare adjusts reimbursement amounts at the level of major metropolitan areas and states, making precise regional differences ambiguous [25]. Lastly, using Medicare data and limiting the scope to non-Mohs surgical procedures and to procedures on the trunk, arms, and legs reduces the generalizability of our findings to only a subset of skin cancer patients and procedures. Future studies that use larger data sources, which include patient demographics, diagnoses, and precise procedural selections, could help further elucidate the physician cost conundrum.

We add to the existing literature on phenotyping physician behavior in skin cancer procedures. Dermatologists and surgeons have been associated with higher costs of NMSC procedures [5], and we have previously demonstrated region and years of experience as predictive factors in NMSC procedures [18,24]. Here, we expand the analysis to procedures used to treat all subtypes of skin cancer carried out by several of the top-performing specialties, highlighting a stark difference in costs between physician cost phenotypes. Furthermore, we use a novel method, cluster analysis, to identify physician risk factors for high removal and closure costs, highlighting a path toward actionable physician improvement and cost reductions similar to the Improving Wisely initiative [8]. Large-scale data could thus be used to provide individual physician-level feedback, identifying outliers based on relative costs by the procedure type and frequency of more complex surgical modality utilization. As with Improving Wisely’s focus on educational, non-punitive measures, this feedback would be aimed at encouraging such outliers to look to peers or to published guidelines to seek actionable methods to optimize care, both medically and financially, for patients.

## Conclusions

This study explored individual physician-level factors associated with higher costs of skin cancer procedures. In aggregate, dermatologists had the highest mean costs per removal and per closure; they also had higher-than-expected total removal costs. Surgeons had higher total closure costs but lower per-procedural costs than the mean. After adjusting for procedural type and volume, removal and closure cost differences were analyzed with k-means clustering, which found four physician cost phenotypes. In cluster analysis, male gender and Northeastern, Western, and wealthy practice location predict high removal costs. More experience and higher MHI predict high costs for both removal and closure. Conversely, practice in the Midwest is associated with lower costs for removals and closures, and dermatology is associated with lower closure costs. This study is limited by the lack of specific diagnoses and facility-based fees, the absence of patient-specific data and outcomes, and the exclusion of Mohs procedures. Identifying factors that predict physicians’ costs for skin cancer treatment may aid efforts to reduce undue economic burden on Medicare patients and to improve physician performance.
